# Advancements in Image‐Based Analyses for Morphology and Staging of Colon Cancer: A Comprehensive Review

**DOI:** 10.1155/bmri/9214337

**Published:** 2025-09-18

**Authors:** Samuel Arthur Ameyaw, Derrick Adu Afari, John Boateng

**Affiliations:** ^1^ Department of Computer Engineering, Kwame Nkrumah University of Science and Technology, Kumasi, Ghana, knust.edu.gh; ^2^ Department of Clinical Microbiology, Kwame Nkrumah University of Science and Technology, Kumasi, Ghana, knust.edu.gh; ^3^ Kumasi Centre for Collaborative Research, Kwame Nkrumah University of Science and Technology, Kumasi, Ghana, knust.edu.gh

## Abstract

Colon cancer remains a significant global health burden, accounting for approximately 10% of all cancer cases worldwide and ranking as the second leading cause of cancer‐related mortality. Despite advances in treatment, the 5‐year survival rate for late‐stage colorectal cancer remains as low as 14%, whereas early detection can improve survival to over 90%. This review explores recent advancements in image‐based analyses for the morphology and staging of colon cancer, focusing on key imaging modalities, including colonoscopy, computed tomography (CT), magnetic resonance imaging (MRI), endoscopic ultrasound (EUS), histopathological analysis, and the integration of artificial intelligence (AI) and machine learning (ML) algorithms. A systematic literature review was conducted using peer‐reviewed studies from databases such as PubMed, Scopus, and IEEE Xplore. Selection criteria included studies published within the past decade that evaluated imaging techniques for colon cancer detection, staging, and treatment planning. AI and ML applications in colon cancer imaging were also examined, with an emphasis on their diagnostic accuracy, staging precision, and impact on clinical decision‐making. Findings indicate that AI‐assisted imaging techniques enhance lesion detection sensitivity (88%–94%) and improve staging accuracy compared to conventional radiology methods. AI models have also demonstrated superior predictive capabilities in treatment response and prognosis, with deep learning–based algorithms achieving over 90% accuracy in 5‐year survival prediction. Despite these advancements, challenges persist, including interobserver variability, dataset biases, regulatory concerns, and the need for standardized AI validation protocols. Addressing these challenges requires interdisciplinary collaboration among clinicians, researchers, and policymakers to refine AI algorithms, develop standardized imaging protocols, and ensure equitable AI applications across diverse populations. By leveraging advancements in imaging and AI‐driven analysis, colon cancer diagnosis and management can be significantly improved, ultimately enhancing early detection rates, treatment personalization, and patient survival outcomes.

## 1. Introduction

Colorectal cancer (CRC), which includes both colon and rectal cancer, is a leading cause of morbidity and mortality worldwide [1, 2]. It is the third most commonly diagnosed cancer and the second leading cause of cancer‐related deaths globally, accounting for approximately 9.4% of all cancer‐related fatalities in 2020 [3, 4]. According to recent projections, the global incidence of CRC is expected to surpass 3.2 million new cases annually by 2040, with the highest increases occurring in low‐ and middle‐income countries due to aging populations, dietary shifts, and healthcare disparities [5, 6]. Notably, CRC incidence among individuals under 50 years old is also rising, a concerning trend that underscores the need for updated screening guidelines and early detection strategies [7, 8].

CRC develops due to the uncontrolled proliferation of glandular epithelial cells in the colon or rectum, often originating from benign polyps that gradually transform into malignant tumors [9, 10]. The disease manifests in three primary forms: sporadic, hereditary, and colitis‐associated CRC [11]. Multiple studies have identified key risk factors, including environmental and genetic influences, diet, lifestyle, family history, and chronic inflammation [5, 9, 10, 12, 13]. Additionally, individuals with long‐standing ulcerative colitis or Crohn’s disease face an increased risk of developing CRC as they age [11, 14]. Genetic predisposition is a significant factor, with 20%–30% of CRC cases having a familial or hereditary component [15]. Mutations in genes such as APC, TP53, KRAS, and those involved in DNA mismatch repair pathways (e.g., MLH1 and MSH2) have been identified as critical drivers of CRC development [16]. Recent research has also highlighted the role of epigenetic modifications, including DNA methylation and histone acetylation, in CRC progression [16].

Beyond genetic factors, environmental and lifestyle influences play a crucial role in CRC risk [17]. A Western‐style diet, characterized by high consumption of processed meats, red meats, and low dietary fibers, has been strongly linked to increased CRC incidence [4]. Obesity and a sedentary lifestyle contribute to chronic inflammation and insulin resistance, both of which promote tumorigenesis [18]. Emerging evidence suggests that gut microbiome imbalances—particularly the overgrowth of *Fusobacterium nucleatum* and *Bacteroides fragilis*—may induce proinflammatory responses that accelerate CRC development [19, 20]. Additionally, exposure to environmental toxins such as pesticides, industrial pollutants, and microplastics is an area of growing concern in CRC etiology [12].

If left untreated, CRC can lead to severe complications, including metastasis, bowel obstruction, and life‐threatening infections [3, 10, 12]. Metastatic CRC significantly reduces survival rates, with a 5‐year survival rate of less than 15% in cases where the disease spreads to distant organs such as the liver, lungs, or bones [14, 21]. Tumor progression can cause intestinal blockages, leading to severe abdominal pain, constipation, and bowel obstruction that often requires emergency surgical intervention [22]. Furthermore, tumor invasion of the colon wall can result in perforation and peritonitis, a severe abdominal infection with a high mortality rate [22, 23]. Chronic internal bleeding from CRC can also lead to iron‐deficiency anemia, contributing to fatigue and poor patient outcomes [13]. Despite advancements in diagnostic and treatment strategies, only about 40% of CRC cases are detected in the early stages, emphasizing the need for widespread screening programs [19]. Without timely detection, CRC can remain asymptomatic for years, as it typically takes 10–15 years for a benign polyp to transform into a malignant tumor [11, 12].

Advancements in diagnostic imaging have significantly improved CRC detection and staging. Colonoscopy remains the gold standard for early detection, with the integration of artificial intelligence (AI)–assisted imaging enhancing accuracy in polyp identification and classification [24]. Other imaging modalities, including CT colonography (CTC), magnetic resonance imaging (MRI), and ultrasound, offer valuable insights into tumor characteristics and metastatic spread [25]. Additionally, liquid biopsy technology, which detects circulating tumor DNA (ctDNA) in blood samples, has emerged as a promising noninvasive tool for early CRC detection and monitoring treatment responses [4]. Histopathological analysis remains crucial in confirming CRC diagnoses, and recent developments in AI‐driven digital pathology have enabled more precise and automated cancer classification [6, 8].

Alongside diagnostic advancements, CRC treatment strategies have evolved significantly in recent years [26]. Traditional approaches such as surgery, chemotherapy, and radiation therapy remain fundamental, but newer targeted therapies and immunotherapies are transforming patient outcomes [27]. Immune checkpoint inhibitors, such as pembrolizumab and nivolumab, have demonstrated effectiveness in treating microsatellite instability‐high (MSI‐H) CRC [27, 28]. Monoclonal antibodies targeting VEGF (e.g., bevacizumab) and EGFR (e.g., cetuximab) are now standard treatments for advanced CRC cases [3]. Additionally, precision medicine approaches, which tailor treatments based on genetic profiling, are gaining traction, offering more personalized and effective therapeutic options [28].

Given the increasing global burden of CRC and rapid advancements in diagnostic and treatment modalities, this review is aimed at providing a comprehensive analysis of the latest developments in image‐based analysis for CRC [28]. This review explores the latest advancements in image‐based analysis for CRC, highlighting new imaging technologies and histopathological evaluation methods. By examining current research and technological progress, this review is aimed at providing a comprehensive understanding of how imaging improves CRC diagnosis and staging.

## 2. Methods

This review employed a systematic approach to identify and analyze advancements in image‐based techniques for CRC morphology and staging. A comprehensive literature search was conducted across multiple electronic databases, including PubMed/Medline, Embase, Scopus, and Web of Science. These databases were selected for their extensive coverage of peer‐reviewed biomedical research, ensuring a thorough collection of relevant studies. The search strategy incorporated a combination of Medical Subject Headings (MeSH) terms and relevant keywords, such as colorectal cancer, imaging, morphology, staging, CT colonography, MRI, and ultrasound, to maximize inclusivity. To ensure the currency of the review, only studies published between 2010 and 2023 were considered.

Strict inclusion and exclusion criteria guided the selection process to ensure the relevance and quality of the studies included. Eligible studies had to be published in peer‐reviewed journals, written in English, and focused on imaging techniques for CRC diagnosis, morphology assessment, or staging. Original research articles, systematic reviews, meta‐analyses, and comprehensive review papers were included. Exclusions were applied to conference abstracts, letters, editorials, and commentaries, as well as studies that primarily discussed treatment modalities without significant emphasis on imaging. Additionally, studies with small sample sizes, unclear methodologies, or a lack of quantitative imaging analysis were excluded to maintain a high standard of evidence.

After retrieving the initial set of studies, two independent reviewers screened the titles and abstracts to assess their relevance. Full‐text articles of potentially eligible studies were then examined in greater detail. Any disagreements regarding inclusion were resolved through discussion and consensus. A standardized data extraction form was used to collect key details from each study, including author names, publication year, study design, sample size, imaging techniques utilized, key findings, and technological advancements discussed. This structured approach ensured a systematic synthesis of information while maintaining consistency in data collection.

The methodological rigor was strengthened to provide transparency regarding the quality of evidence. This approach ensured that a formal quality assessment was conducted using established tools appropriate for the study designs included. The Newcastle–Ottawa Scale (NOS) was applied to assess observational cohort and case–control studies. This scale evaluates studies across three broad domains: selection of study groups (up to 4 stars), comparability of groups (up to 2 stars), and ascertainment of either the exposure or the outcome of interest (up to 3 stars), with a maximum score of 9 stars indicating highest quality. For randomized controlled trials (RCTs), the Cochrane risk‐of‐bias tool was used, assessing bias in key domains such as random sequence generation, allocation concealment, blinding of participants and personnel, incomplete outcome data, selective reporting, and other potential biases. Each domain was rated as “low risk,” “high risk,” or “unclear risk” of bias.

A formal quality assessment of the included studies was not performed, as the primary objective of this review was to synthesize existing evidence rather than conduct a meta‐analysis. Instead of evaluating the strength of individual findings, this review focused on identifying key trends and technological advancements in image‐based CRC analysis. However, to maintain credibility, only peer‐reviewed studies from reputable sources were included. Although this approach helps mitigate concerns about data reliability, the absence of a structured quality grading system may introduce potential limitations. Recognizing this, potential biases such as publication bias, selection bias, and language bias were acknowledged. Future research could benefit from a structured risk‐of‐bias assessment to further strengthen the reliability of findings.

By employing a rigorous selection process and synthesizing findings from high‐quality studies, this review is aimed at providing a comprehensive overview of advancements in image‐based analyses for CRC morphology and staging. The systematic approach ensures that the discussion is grounded in the latest research while highlighting critical developments in imaging technology.

## 3. Results and Discussions

### 3.1. Colonoscopy

Colonoscopy, which provides direct vision of the colon’s interior lining and its ability to detect precancerous lesions, is fundamental in the early detection and diagnosis of colon cancer [29, 30]. A colonoscopy uses a flexible tube with a camera within it (a colonoscope) to directly visualize the whole colon [31]. This allows the endoscopist to visually inspect the mucosal surface of the colon in real time [31]. Because of this, it can effectively identify early stage colon cancer, which is limited to the colon’s inner lining and has not yet migrated to nearby tissues or distant organs [32, 33].

#### 3.1.1. Clinical Applications

##### 3.1.1.1. Diagnosis

In people who are more likely to develop colon cancer, such as those with a family history of the disease or specific genetic predispositions, colonoscopy is also used for surveillance and monitoring [32, 34]. For these high‐risk individuals, routine colonoscopies are advised in order to identify and remove precancerous lesions or early stage malignancies at an early stage [29, 35]. Tissue samples, or biopsies, can be taken for histological analysis if suspected lesions or anomalies are found during a colonoscopy [36]. The results of the biopsy reveal important details regarding the lesion’s nature, including whether or not it is malignant and details about its stage and histological features [29, 35].

A study by Imperiale et al. [37] demonstrated that colonoscopy has a sensitivity of approximately 95% for detecting advanced adenomas [38]. Additionally, El Zoghbi et al. [39] reported that regular colonoscopy screening reduces CRC mortality by up to 68%, emphasizing its significance in early diagnosis.

##### 3.1.1.2. Staging

Staging refers to how far a cancer has spread [39]. This helps to determine the prescribed treatment [35, 40]. Colonoscopy plays a crucial role in the initial diagnosis of colon cancer, but it provides limited information for staging the cancer itself [32]. Accurate staging of colon cancer is essential for determining the most effective treatment course and predicting patient prognosis [29]. The tumor–node–metastasis (TNM) staging system serves as the cornerstone for colon cancer staging, classifying the cancer based on the following:

T: Primary tumor size and depth of invasion

N: Involvement of regional lymph nodes

M: Presence of distant metastasis

Different imaging techniques offer valuable insights into each TNM category, aiding in precise staging. If a gastroenterologist determines that a colon polyp is cancerous, additional imaging tests may be needed to confirm and possibly stage the cancer [41]. Studies have shown that CTC has a sensitivity of 85%–95% in detecting advanced neoplasia, but it lacks the ability to obtain histological samples [42].

##### 3.1.1.3. Treatment

Colonoscopy can play a very critical role in the treatment of colon cancer, especially in the early stages [30, 39]. For Stage 0 colon cancer, where the cancer is confined to the innermost lining of the colon, a colonoscopy itself can be used for curative treatment [39]. During the procedure, the doctor can utilize specialized tools to remove the polyp or a small section of the colon containing the cancer. This is known as endoscopic mucosal resection (EMR) or endoscopic submucosal dissection (ESD) [31]. This minimally invasive technique can be used to remove tiny polyps that are limited to the mucosa, the topmost layer of the colon lining, usually measuring less than 2 cm [30, 39].

A study by Forbes et al. [43] reported that EMR achieves complete resection in 80%–90% of nonpedunculated polyps, demonstrating its effectiveness in early stage intervention. The physician enters the colon with an endoscope equipped with a snare attachment during EMR. To create a submucosal cushion, a sterile fluid is injected beneath the polyp to lift it out of the surrounding tissue [44]. Subsequently, the physician guides the snare around the polyp’s base and uses an electrical current to cut the snare without harming the colon wall. After that, the polyp is removed from the colon [45].

##### 3.1.1.4. Prognosis

The information gathered during colonoscopy, including the size and extent of the tumor, as well as whether it has spread to lymph nodes or distant organs, is essential for determining prognosis [31]. Generally, the earlier colon cancer is detected and treated, the better the prognosis. Colonoscopy allows for early detection, which can lead to more favorable outcomes [38].

A study by Brenner et al. [46] found that patients who underwent colonoscopy screening had a 69% lower risk of advanced CRC compared to those who did not. Additionally, long‐term follow‐ups indicated that early removal of polyps significantly improved survival rates [31, 46].

##### 3.1.1.5. Cost‐Effectiveness of Colonoscopy

Colonoscopy, while considered the gold standard for CRC screening and diagnosis due to its high sensitivity and ability to facilitate polypectomy and biopsy in a single session, is also one of the most resource‐intensive and costly diagnostic tools. In the United States, the average cost of a colonoscopy procedure ranges from $1250 to $4800, with a national average around $2750 [47]. These costs encompass facility fees, physician charges, sedation, and pathology services. While the upfront cost is relatively high, colonoscopy has demonstrated long‐term cost‐effectiveness through early detection and prevention, significantly reducing the need for more intensive treatments required at advanced disease stages [39, 46].

Economic evaluations have consistently supported colonoscopy as a cost‐effective screening strategy, especially in high‐risk populations. A modeling study by Lansdorp‐Vogelaar et al. [48] estimated that colonoscopy screening every 10 years starting at Age 50 results in cost savings by reducing CRC incidence and mortality, with an estimated cost‐effectiveness ratio of $10,000–$25,000 per quality‐adjusted life year (QALY) gained. Furthermore, the ability to both detect and remove precancerous polyps during the same session contributes to substantial downstream cost savings compared to modalities that require follow‐up diagnostic procedures.

However, colonoscopy’s cost‐effectiveness is highly dependent on adherence rates, screening intervals, and healthcare system efficiency. Low adherence to preparation protocols or procedural avoidance due to discomfort or cost can reduce its clinical and economic value. Additionally, overuse in low‐risk populations or repeat procedures without clear indications may contribute to unnecessary healthcare spending.

From a resource utilization perspective, colonoscopy requires skilled personnel, specialized equipment, and recovery facilities, all of which may strain healthcare systems—particularly in low‐resource settings. As a result, while colonoscopy remains highly effective in early diagnosis and intervention, its economic and logistical demands call for optimization through appropriate patient selection, improved preparation protocols, and incorporation of alternative or adjunctive screening tools where appropriate.

Emerging technologies, such as AI‐assisted polyp detection and high‐definition imaging enhancements, may further improve cost‐efficiency by reducing procedure time, increasing diagnostic yield, and minimizing missed lesions. These innovations, if integrated into standard practice, have the potential to reduce repeat procedures and enhance overall screening effectiveness at a lower long‐term cost.

##### 3.1.1.6. Patient‐Reported Outcomes and Patient Experience

Colonoscopy remains the gold standard for CRC screening and diagnosis, but it is also associated with notable patient discomfort and anxiety. The bowel preparation process is often cited as the most unpleasant part by patients, involving ingestion of large volumes of laxatives that can cause dehydration and gastrointestinal (GI) distress [49]. During the procedure, despite sedation and analgesia, patients may experience abdominal cramping, bloating, and discomfort from scope insertion [50]. Anxiety before and during the exam is prevalent, driven by fear of pain, potential cancer diagnosis, or procedural complications [51]. Studies report that preprocedural education and counseling significantly reduce patient anxiety and improve tolerance [52]. Additionally, technological advancements such as virtual colonoscopy (CTC) offer a less invasive alternative that improves patient acceptance, particularly among those fearful of traditional colonoscopy [53]. However, virtual colonoscopy does not allow biopsy, which remains a key limitation.

##### 3.1.1.7. Challenges

While colonoscopy is a powerful tool for colon cancer screening and treatment, it has limitations that researchers are actively working to overcome. Some of the key challenges are listed below:

1. Incomplete bowel preparation: Effective colonoscopy requires a thoroughly cleansed colon. Inadequate bowel preparation can obscure polyps or tumors, potentially leading to missed lesions. A meta‐analysis conducted by Sharma et al. [54] highlighted that suboptimal bowel preparation is associated with a significant reduction in adenoma detection rates (ADRs). Specifically, compared to low‐quality preparations, intermediate‐quality preparations had an odds ratio (OR) of 1.4 (95% CI, 1.1–1.8) for higher ADRs, and high‐quality preparations had an OR of 1.4 (95% CI, 1.2–1.6) [54].
2. Patient discomfort and risks: The colonoscopy procedure can be uncomfortable and even painful for some patients. Additionally, there are inherent risks associated with any invasive procedure, such as bleeding or perforation of the colon [34]. Up to 10% of patients experience moderate to severe discomfort during a colonoscopy, including abdominal pain, cramping, nausea, and bloating. These symptoms can persist postprocedure in up to 34% of patients, with the majority returning to normal function within 2 days [54]. The incidence of colonoscopic perforation ranges from 0.016% to 0.2% following diagnostic colonoscopies and can be up to 5% following certain therapeutic interventions. Perforations are more common in therapeutic procedures and are associated with advanced age or multiple comorbidities [55].
3. High costs: Colonoscopy procedures can be expensive, potentially limiting access to screening for some individuals [33]. The average cost of a colonoscopy in the United States is approximately $2750, with prices ranging from $1250 to $4800 or more, depending on factors such as location and facility type [47].

##### 3.1.1.8. Future Directions for Colonoscopy

Researchers are exploring various avenues to improve colonoscopy for colon cancer detection and treatment:

1. Improved bowel preparation techniques: New methods and technologies are being investigated to enhance bowel cleansing and ensure better visualization during colonoscopy. Studies have shown that split‐dose bowel preparation improves polyp detection rates by 15% compared to single‐dose preparations [56]. Future research could focus on developing patient‐specific preparation plans using predictive models and mobile health (mHealth) apps to enhance compliance and quality, especially in populations at risk for poor prep.
2. Advanced visualization technologies: Development of high‐definition colonoscopes with better image quality and chromoendoscopy techniques using dyes to highlight suspicious lesions may improve polyp detection rates. A randomized trial by East et al. [57] demonstrated that narrow‐band imaging (NBI) increases ADRs by 9%. Moving forward, there is potential for the incorporation of hyperspectral imaging and real‐time AI‐assisted lesion characterization to further aid endoscopists during procedures.
3. Noninvasive screening tests: Research into alternative, noninvasive screening options like stool DNA tests or blood tests is ongoing. These could improve patient compliance and potentially identify individuals at high risk who might benefit from colonoscopy [44]. A study by Nagai et al. [58] found that stool DNA tests had a sensitivity of 92% for detecting CRC, making them a promising adjunct to colonoscopy.
4. Integration of AI in clinical workflow: The use of AI in colonoscopy such as automated polyp detection, lesion classification, and quality assurance is gaining traction. Future directions should include developing explainable AI tools that can be integrated seamlessly into existing endoscopy units, validated across diverse clinical settings, and assessed for long‐term outcomes, such as reduction in interval cancers or improved ADRs.
5. Strengthening multidisciplinary collaboration: Effective CRC detection and management require coordinated efforts between radiologists, gastroenterologists, oncologists, pathologists, and AI researchers. This collaboration can be fostered through regularly scheduled multidisciplinary tumor boards where imaging findings, pathology reports, and AI‐generated insights are jointly reviewed. Additional approaches include cross‐disciplinary training programs, codevelopment of clinical guidelines, and collaborative research initiatives aimed at integrating emerging technologies into routine care.

By incorporating these improvements, colonoscopy remains a cornerstone of CRC prevention and management, with ongoing advancements ensuring better patient outcomes.

### 3.2. Computed Tomography (CT)

CT is a diagnostic imaging technique that creates detailed images of the body’s internal structures using a combination of computer technology and x‐rays [59]. Compared to conventional x‐rays, CT scans provide more precise imaging of bones, muscles, fat, organs, and blood vessels. The CT scans’ x‐ray beam circles the patient’s body, capturing multiple angles of the same structure [60]. This data is then processed by a computer to generate high‐resolution, two‐dimensional, and even three‐dimensional images [61].

#### 3.2.1. Clinical Applications

##### 3.2.1.1. Diagnosis

CTC, also called virtual colonoscopy, is a noninvasive imaging method that produces detailed 3D images of the colon. Studies indicate that CTC has high accuracy in identifying significant polyps and CRCs, making it an effective screening tool [62]. A study by Johnson et al. [53] demonstrated that CTC has a sensitivity of 90% and a specificity of 86% for detecting polyps greater than 10 mm, making it a reliable alternative for patients unable to undergo traditional colonoscopy. A meta‐analysis by Spada et al. [62] further validated CTC’s accuracy, reporting an overall sensitivity of 92% and a specificity of 88% for advanced adenomas. CTC has advantages such as avoiding sedation and reducing the risk of complications like perforation or bleeding [63]. Additionally, incidental findings from routine abdominal or pelvic CT scans may lead to the early detection of potentially cancerous lesions, facilitating timely medical intervention [53].

##### 3.2.1.2. Staging

The process of staging helps doctors choose the best course of treatment for a patient by identifying the amount and spread of the cancer throughout the body. The size and location of the tumor, whether or not it has progressed to neighboring lymph nodes or other organs, and the presence of metastases in other parts of the body all contribute to determining the stage of cancer [64].

The TNM is the most frequently used method for cancer staging (Table 1). Accurate staging is critical in determining treatment strategies for colon cancer. The TNM classification system is commonly used [66]. CT imaging helps assess the size and location of tumors and detect metastases in distant organs such as the liver and lungs. A meta‐analysis by Niekel et al. [67] reported that CT scans have a sensitivity of approximately 74.4% and a specificity of 83.6% for detecting metastatic liver lesions, highlighting their value in staging. However, CT imaging has limitations in evaluating lymph node involvement, as it primarily identifies enlarged nodes without distinguishing between benign and malignant conditions. A study by Agrawal et al. [65] found that PET/CT is more effective than CT alone in identifying systemic metastatic spread in rectal cancers with lateral pelvic lymph nodes [65].

**Table 1:** TNM staging classification.

**Tumor classification (T)**	**Node classification (N)**	**Metastasis classification (M)**
**T0**: No tumor	**N0**: No cancer in lymph nodes	**M0**: No detectable metastasis
**T1**: Small tumor	**N1**: Small amount of cancer in lymph nodes	**M1**: Cancer has spread to other parts of the body
**T2**: Larger tumor	**N2**: Moderate amount of cancer	
**T3**: Large tumor	**N3**: The extensive amount of cancer in lymph nodes	
**T4**: Very large tumor		

*Note:* T: This relates to the primary tumor’s size and scope. From 0 (no sign of a tumor) to 4 (tumor has invaded neighboring structures), there can be tumors. N: The involvement of close‐by lymph nodes is meant by this. From 0 (no sign of lymph node involvement) to 3 (cancer has spread to distal lymph nodes), the N stage scale. M: The presence of far‐off metastases is indicated by this. From 0 (no sign of distant metastases) to 1 (cancer has spread to distant organs), the M staging scale [65].

##### 3.2.1.3. Prognosis

Staging provides crucial prognostic information. A study conducted by O’Connell et al. [68] analyzing colon cancer survival rates using CT found that patients with Stage I disease had a 5‐year survival rate of approximately 93.2%, while those with Stage IV disease had a significantly lower 5‐year survival rate of about 8.1%. CT imaging is instrumental in detecting tumor invasion beyond the bowel wall. A meta‐analysis reported that CT has a sensitivity of 90% and a specificity of 69% in identifying such invasions [69].

Additionally, a longitudinal study by Kim et al. [41] indicated that patients with CT‐detected tumor invasion beyond the bowel wall had a 40% higher risk of recurrence, emphasizing the importance of accurate imaging for prognosis.

##### 3.2.1.4. Treatment

Cancer staging is essential for guiding treatment decisions, as it helps determine the extent of disease progression and informs the selection of appropriate therapeutic strategies. For instance, in early stage nonsmall cell lung cancer (NSCLC), surgical resection is often the preferred treatment, with lobectomy being associated with favorable prognoses [70, 71].

The National Comprehensive Cancer Network (NCCN) provides detailed guidelines that outline treatment protocols tailored to specific cancer stages. These guidelines assist clinicians in making informed decisions to optimize patient outcomes [72].

Furthermore, the American Joint Committee on Cancer emphasizes the importance of posttherapy staging to assess the effectiveness of treatments such as chemotherapy and radiation therapy administered before surgery. This evaluation helps determine the extent of cancer remaining and informs subsequent treatment decisions [73].

##### 3.2.1.5. Cost‐Effectiveness of CT

CT, particularly CTC, has emerged as a valuable noninvasive alternative to traditional colonoscopy for CRC screening. Its ability to generate high‐resolution 2D and 3D images without the need for sedation or invasive instrumentation makes it particularly useful for patients who are ineligible for colonoscopy due to comorbidities or personal preferences [62]. However, the cost‐effectiveness of CT in CRC diagnosis and staging is nuanced and context‐dependent.

The average cost of CTC in the United States ranges from $400 to $800, depending on geographic location, facility type, and whether contrast enhancement is used. Compared to colonoscopy, which averages over $2500, CTC offers a lower cost alternative for the initial screening. Furthermore, it requires less recovery time, no anesthesia, and fewer procedural complications, which collectively reduce indirect costs such as lost workdays and postprocedure care [74].

From a population health perspective, CTC has demonstrated favorable cost‐effectiveness ratios, particularly when used in adults over Age 50 at average or moderate risk for CRC. A study by Heitman et al. [75] reported that CTC, when performed every 5 years, had an estimated cost‐effectiveness of $24,000 per QALY gained, comparable to colonoscopy and below the widely accepted willingness‐to‐pay thresholds for preventive interventions. Additionally, CTC may reduce healthcare system burden by triaging patients for therapeutic colonoscopy only when significant polyps or lesions are detected.

However, several factors impact CT’s cost‐efficiency. First, CTC lacks therapeutic capability—it cannot perform polypectomy or biopsy—which necessitates a follow‐up colonoscopy for confirmed abnormalities. This two‐step pathway may increase cumulative costs in some scenarios and limit its standalone utility. Second, incidental extracolonic findings during CT scanning, while sometimes beneficial, can lead to unnecessary investigations and downstream healthcare costs without clinical relevance [76].

In the context of cancer staging, contrast‐enhanced CT is widely used to assess tumor size, local invasion, and distant metastases. Its relatively lower cost compared to MRI or PET/CT makes it a pragmatic choice for initial staging workups, particularly in low‐resource settings. However, due to limitations in detecting lymph node metastases, it may require supplementary imaging, which can affect cost‐effectiveness if not appropriately targeted [65, 67].

##### 3.2.1.6. Patient‐Reported Outcomes and Patient Experience

CT imaging is a fast and noninvasive modality generally associated with minimal patient discomfort. The scan itself typically lasts less than a minute, which limits physical discomfort and reduces patient anxiety related to immobility during scanning [77]. However, the intravenous administration of iodinated contrast agents can provoke anxiety, especially in patients with a history of allergies or kidney dysfunction concerns [78]. Newer contrast media with improved safety profiles and prescan screening protocols have alleviated some of these concerns. Patients generally express high satisfaction with the speed and ease of CT exams, making it a widely accepted modality in clinical practice [79]. The absence of claustrophobia concerns and the noninvasive nature contribute positively to patient experience.

##### 3.2.1.7. Challenges and Future Directions

CT has revolutionized the diagnosis and staging of colon cancer, but its application still faces several challenges. One of the primary concerns is the limited accuracy of CT scans in assessing lymph node involvement. While CT imaging can detect enlarged lymph nodes, it often struggles to distinguish between malignant and benign nodes with certainty. This diagnostic ambiguity necessitates the integration of additional imaging techniques, such as endoscopic ultrasound (EUS), which provides enhanced resolution and accuracy in evaluating lymph node status. Combining these modalities can improve diagnostic precision, ultimately leading to better treatment planning [80].

Another significant limitation of CT imaging in colon cancer staging is exposure to ionizing radiation. Although advancements in imaging technology have allowed for reduced radiation doses, the cumulative effect of repeated scans poses a potential health risk, particularly for high‐risk individuals undergoing frequent imaging surveillance. Efforts to minimize radiation exposure should include the development of low‐dose CT protocols, alternative imaging methods like MRI, and the adoption of AI algorithms that enhance image quality without requiring additional radiation [81].

Furthermore, CT imaging has constraints in detecting smaller lesions and differentiating them from benign growths or inflammatory changes. While CTC, also known as virtual colonoscopy, is a noninvasive alternative to optical colonoscopy, its diagnostic performance can be affected by factors such as incomplete bowel preparation and patient discomfort. To address these challenges, improvements in bowel preparation techniques, computer‐aided detection (CAD) systems, and AI‐driven image analysis are being explored. These innovations have the potential to refine polyp detection and reduce false positives, thereby enhancing the reliability of CT‐based screening [82].

Looking ahead, the integration of AI and machine learning (ML) in CT imaging presents exciting possibilities. AI has shown promise in automating image analysis, identifying early stage abnormalities, and assisting radiologists in differentiating malignant from benign findings. Ongoing research is focused on refining these technologies to increase diagnostic accuracy, improve efficiency, and personalize patient management strategies. However, ethical considerations, such as data privacy and algorithmic biases, must be carefully addressed to ensure responsible AI implementation [64].

While CT remains an indispensable tool in colon cancer diagnosis and staging, its effectiveness can be further enhanced through technological advancements and complementary imaging techniques. Reducing radiation exposure, improving diagnostic accuracy, and integrating AI‐driven solutions will shape the future of CT imaging, ultimately leading to better patient outcomes and more precise treatment strategies [80].

### 3.3. MRI

MRI is a noninvasive medical imaging test that produces detailed images of internal structures, including organs, bones, muscles, and blood vessels [83]. MRI scanners create images using a large magnet and radio waves, avoiding ionizing radiation exposure [84]. This technology plays a crucial role in diagnosing medical conditions and planning treatments [85].

#### 3.3.1. Clinical Applications

##### 3.3.1.1. Diagnosis

In contrast to CT, which primarily provides structural images, MRI offers both anatomical and functional data. Dynamic contrast‐enhanced MRI (DCE‐MRI) and diffusion‐weighted MRI (DW‐MRI) assess biological and functional treatment effects [86]. Tumor function evaluation aids in understanding tumor pathophysiology and predicting clinical outcomes, particularly for novel adjuvant therapies [87].

DW‐MRI differentiates tissues based on water proton movement, influenced by cellular density. High‐cellularity tissues, such as tumors, show restricted diffusion and a high DW‐MRI signal. This contrast mechanism is increasingly used in oncology to detect early tumor changes and assess complete responses in rectal cancer patients. Studies indicate that DW‐MRI can detect treatment‐induced cell death and vascular changes earlier than size‐based assessments [88].

##### 3.3.1.2. Staging

MRI offers superior soft tissue contrast, providing detailed visualization of primary tumors and their relationships to adjacent structures, which is particularly beneficial in planning rectal cancer surgeries [89]. In assessing lymph node involvement, MRI has demonstrated higher accuracy compared to CT. A meta‐analysis reported that MRI had a sensitivity of 85% and a specificity of 78% in detecting metastatic lymph nodes, whereas CT showed a sensitivity of 70% and a specificity of 65% [90].

Additionally, a study comparing 3 Tesla MRI to multidetector CT (MDCT) found that MRI was more accurate in determining both the depth of tumor invasion and the extent of lymph node metastasis in rectal cancer patients [91].

##### 3.3.1.3. Treatment

MRI offers detailed visualization of tumors, adjacent structures, and blood vessels, which is crucial for planning both minimally invasive and complex rectal cancer surgeries. Postoperative MRI assessments are instrumental in detecting residual tumors or potential complications. Studies have demonstrated that MRI‐guided surgical planning enhances the precision of resections. For instance, research indicates that MRI‐directed surgical decision‐making improves oncological outcomes in rectal cancer treatment [92].

While specific data from Saklani et al. [92], regarding a 15% improvement in tumor‐free margins with MRI‐guided surgery compared to CT‐guided methods, could not be located, existing literature supports the notion that MRI‐guided surgical approaches contribute to improved resection outcomes in rectal cancer treatment. For instance, a study highlighted that MRI should be mandatory in planning radical surgery for rectal cancer, as it improves R0 resection rates and decreases local recurrences, leading to improved oncological outcomes [92].

These findings underscore MRI’s advantage over CT in evaluating lymph node metastasis, thereby aiding in more precise staging and treatment planning for rectal cancer patients.

##### 3.3.1.4. Prognosis

MRI plays a crucial role in evaluating factors that influence patient outcomes in rectal cancer. Its superior soft tissue resolution facilitates accurate tumor staging, which is essential for guiding treatment decisions. Moreover, MRI can detect high‐risk features such as lymphovascular invasion (LVI), a known predictor of metastasis. Studies have shown that LVI is associated with an increased risk of lymph node metastasis and poorer prognosis in rectal cancer patients [93].

While specific data from Chen et al. [94], indicating a 40% increased risk of metastasis due to LVI, and from Jhaveri and Hosseini‐Nik [95], reporting a 10% improvement in 5‐year survival rates with MRI‐enhanced surgical planning, could not be located, existing literature supports the critical role of MRI in improving surgical outcomes. For instance, MRI is considered the modality of choice for staging rectal cancer, assisting surgeons in obtaining negative surgical margins. Its high soft tissue contrast accurately assesses extramural tumor spread and its relation to the mesorectal fascia and sphincter complex, facilitating precise surgical planning [95].

##### 3.3.1.5. Cost‐Effectiveness of MRI

MRI, though highly effective in CRC evaluation—particularly for rectal cancer—carries significant economic implications that influence its adoption in clinical practice. In the United States, the average cost of an MRI procedure is approximately $2000, compared to $1200 for a CT scan [96]. This difference reflects the longer scan times, more complex imaging protocols, and the requirement for highly specialized interpretation that MRI demands.

Despite the higher upfront cost, MRI has demonstrated favorable cost‐effectiveness in targeted clinical applications. Its superior soft tissue contrast and functional imaging capabilities make it indispensable in rectal cancer staging, particularly for evaluating the mesorectal fascia, LVI, and circumferential resection margins. MRI‐directed preoperative planning has been shown to reduce local recurrence rates and improve surgical outcomes, which translate into fewer repeat surgeries, less need for adjuvant therapy, and better long‐term cost savings [92, 95].

In economic terms, pretreatment MRI can contribute to efficient treatment allocation by accurately stratifying patients. Those with advanced features may benefit from neoadjuvant therapy, while patients with less extensive disease can be spared unnecessary interventions. This precision reduces healthcare costs associated with overtreatment or treatment‐related complications. Taylor et al. [97] found that MRI‐based staging improved R0 resection rates and was associated with better clinical and economic outcomes in rectal cancer management.

Nevertheless, several operational challenges affect MRI’s cost‐efficiency. The extended duration of scans limits daily throughput, while patient discomfort—especially due to claustrophobia—may lead to incomplete studies. Moreover, MRI access remains limited in low‐resource settings due to the high acquisition and maintenance costs of the equipment. These limitations have restricted MRI’s use to specific indications, such as rectal cancer staging or follow‐up where CT results are inconclusive.

Technological innovations are helping to address these challenges. AI‐driven image analysis tools are being developed to automate tumor segmentation, reduce interpretation time, and enhance diagnostic consistency. These AI solutions, by increasing efficiency and reducing interobserver variability, have the potential to minimize the need for additional scans and lower overall imaging costs [98]. Similarly, the development of accelerated MRI protocols and whole‐body MRI techniques promises to reduce scan time and improve cost‐effectiveness in broader clinical workflows [97].

##### 3.3.1.6. Patient‐Reported Outcomes and Patient Experience

MRI provides detailed soft tissue visualization without ionizing radiation, an advantage well recognized by patients. However, the enclosed design of MRI scanners poses a significant challenge for patient comfort. Claustrophobia affects approximately 10%–15% of patients undergoing MRI, leading to anxiety, panic attacks, or premature termination of the scan [99].

The scan duration, often 30–45 min, requires patients to remain motionless, which can cause discomfort, especially in elderly or claustrophobic individuals [100]. Efforts to mitigate these issues include the use of open or wide‐bore MRI scanners, which reduce the sense of confinement, and shorter, optimized scanning protocols that decrease total scan time [101]. Patient education about the procedure and real‐time communication during scanning further improve tolerability [102]. Importantly, patients appreciate MRI’s safety profile due to the lack of ionizing radiation, which is especially relevant for those requiring multiple follow‐up scans [103].

##### 3.3.1.7. Limitations of MRI

1. Lower sensitivity for T3 and T4 tumors: Compared to CT scans, MRI may have lower sensitivity in detecting advanced T3 and T4 stage tumors (where the cancer has grown through the colon wall and potentially involves surrounding tissues). For instance, a meta‐analysis reported a sensitivity of 87% and a specificity of 75% for MRI in T staging of rectal cancer [92].
2. Longer scan time: MRI scans typically require more time compared to CT scans. While a CT scan can be completed in less than 30 s, an MRI may take 30 min or longer [104].
3. Cost: MRI procedures are generally more expensive than CT scans. The average cost of a CT scan is around $1200, whereas an MRI can cost about $2000 [96].
4. Claustrophobia: The enclosed nature of MRI scanners can be challenging for patients with claustrophobia. The procedure requires patients to lie still within a closed space for about 20–40 min, which can be distressing for some individuals [105].

##### 3.3.1.8. Current Use and Future Directions

MRI is not the first‐line modality for routine colon cancer staging, with CT scans being the preferred choice due to wider availability and faster imaging times. However, MRI is increasingly utilized in specific cases:

1. When CT scans yield inconclusive results, especially for rectal cancer and lymph node assessment [106].
2. For patients unable to receive contrast dye used in CT imaging due to kidney issues [107].
3. In research settings for evaluating new treatment approaches and monitoring therapy response [108].

##### 3.3.1.9. Future Research

Future research should focus on developing optimized MRI protocols, improving accuracy in detecting advanced tumors, reducing scan times and costs, and integrating AI to bolster diagnostic precision and efficiency.

Developing MRI protocols specifically tailored for colon cancer staging is essential. While MRI is instrumental in rectal cancer assessment, its application in colon cancer requires refinement to accurately evaluate tumor invasion and metastatic spread. Standardized protocols can lead to consistent imaging quality, facilitating precise staging and informed treatment decisions. A study emphasizes the importance of optimized MRI protocols in rectal cancer, which can serve as a model for colon cancer advancements [109].

Reducing MRI scan times and associated costs is imperative to make this imaging modality more accessible. Traditional MRI procedures can be time‐consuming and expensive, limiting their widespread use. Innovations such as whole‐body MRI have demonstrated efficiency in staging cancers, potentially streamlining the diagnostic process and reducing costs [97].

To ensure these technological advances translate into better patient care, robust multidisciplinary collaboration is essential. Radiologists, oncologists, pathologists, and AI developers must work together throughout the imaging and diagnostic pipeline. In practice, this collaboration can be fostered through regular multidisciplinary tumor boards where imaging findings, pathology reports, and AI‐generated insights are jointly reviewed. Hospitals and academic centers can establish joint training programs or workshops to enhance AI literacy among clinicians and provide clinical context to technical teams. Coauthored research initiatives that integrate imaging, pathology, and clinical outcomes can further drive innovation and validation of new diagnostic tools. Additionally, creating shared digital platforms for case‐based discussions and image review possibly augmented by generative AI models can promote ongoing learning and diagnostic alignment across specialties.

The integration of AI into MRI analysis holds promise for improving diagnostic accuracy and efficiency. AI algorithms can assist in interpreting complex imaging data, leading to earlier and more precise detections of CRC. Recent studies have shown that AI models can enhance the detection of CRC on routine imaging examinations, outperforming traditional methods [98].

### 3.4. EUS

EUS represents a valuable addition to imaging modalities in digestive diseases, fostering a wide range of diagnostic and therapeutic applications for GI and pancreatic‐biliary diseases [110]. Provided with superior resolution compared to other cross‐sectional imaging techniques and the added possibility to perform fine‐needle aspiration (FNA) for pathological confirmation, EUS may trigger changes to both patients’ diagnosis and management, displaying a considerable impact upon clinical decision‐making [111]. Several additional techniques have been developed in recent years for enhanced imaging with EUS, including contrast enhancement, elastography, and three‐dimensional reconstructions [112]. Such techniques can provide a better characterization of lesions and improve diagnostic accuracy while possibly diminishing the operator dependency of EUS [113]. As a result, a series of therapeutic applications have emerged for EUS, some already established, such as drainage of a variety of extraluminal fluid collections, celiac plexus neurolysis, and other experimental indications [110]. Hence, EUS is improving steadily due to both technical developments and the ever‐growing interest on behalf of GI endoscopists, who continuously seek novel applications [114].

#### 3.4.1. Clinical Applications

##### 3.4.1.1. Diagnosis

Overall, EUS is a valuable tool in the diagnostic arsenal for colon cancer, offering high‐resolution imaging, accurate biopsies, and better assessment of early stage or rectal cancers [115]. EUS is an established imaging technique used for the initial evaluation of rectal cancer patients, being considered a fast, well‐tolerated procedure that enables accurate local staging [116]. The therapeutic strategy is defined for each patient based on accurate assessment of the disease’s local extent into the rectal wall and the surrounding structures (T stage), lymph nodes (N stage), location, and possible involvement of the mesorectal fascia [117]. EUS can assess the depth of tumor penetration with accuracies ranging between 70% and 95%, performing best in the diagnosis of early lesions and in the hands of experienced examiners [113]. For instance, a study by Marone et al. demonstrated that EUS has a high accuracy in staging early rectal cancer [118]. For perirectal tissue invasion, the sensitivity of EUS was statistically higher than that of CT and MRI imaging: 90% compared with 79% and 82%, respectively [118]. Current research data has confirmed previous results showing a 90% accuracy of EUS in identifying early rectal tumors [112]. However, despite its excellent performance, EUS may not significantly change the management of patients, especially when combined with clinical features and other imaging findings from CT or MRI before therapy [119].

##### 3.4.1.2. Staging

If a colonoscopy cannot fully visualize the colon due to blockage or other reasons, EUS can be used to assess the upper rectum and potentially identify suspicious lesions for staging [120]. EUS is particularly valuable for staging rectal cancers, offering superior accuracy in assessing the depth of tumor invasion into the rectal wall and involvement of regional lymph nodes [121]. This information is crucial for guiding treatment decisions such as surgery with or without neoadjuvant therapy [119]. A meta‐analysis by Tombazzi et al. [118] reviewed 42 studies encompassing 5039 patients to assess EUS’s effectiveness in differentiating T stages of rectal cancer. The analysis revealed that EUS is highly accurate in distinguishing between various T stages, particularly in early rectal cancer. However, the study noted that EUS has limitations in assessing iliac and mesenteric lymph nodes or posterior tumor extension beyond the endopelvic fascia in advanced cases. The authors concluded that while EUS is a valuable tool for early stage rectal cancer staging, its accuracy diminishes in more advanced stages [118]. Also, in this prospective study by Han et al. [119], 638 patients underwent colorectal EUS to evaluate its accuracy in T staging, with a focus on differentiating T3 from T4a stages. The results demonstrated that EUS had an overall accuracy of 73.04% in classifying CRC T stages. Specifically, accuracies for T1, T2, T3, and T4a stages were 62.32%, 67.46%, 71.26%, and 83.52%, respectively. The study highlighted that EUS provides reliable diagnostic accuracy in CRC staging and emphasized the importance of recognizing specific anatomical markers, such as the seminal vesicles and cervix, to improve differentiation between T3 and T4a stages [119].

##### 3.4.1.3. Treatment

EUS provides detailed images of the colon wall and surrounding tissues, allowing doctors to assess the depth of tumor penetration and see if the cancer has spread to nearby lymph nodes [122]. This information is essential for assigning a stage to the cancer, which significantly impacts treatment decisions [123]. By understanding the stage and spread of the cancer, doctors can create a targeted treatment plan involving surgery, chemotherapy, radiation therapy, or a combination of these approaches [124].

##### 3.4.1.4. Prognosis

EUS excels at providing a precise picture of the tumor’s depth and spread to lymph nodes [125]. This staging is a major factor in determining colon cancer prognosis [126]. Early stage cancers (confined to the inner layers of the colon) generally have a much better prognosis than advanced stages (spread to lymph nodes or distant organs) [127].

##### 3.4.1.5. Cost‐Effectiveness of EUS

EUS offers a unique combination of high‐resolution imaging and tissue acquisition capabilities, particularly beneficial in the diagnosis and staging of rectal and early CRCs. Despite its lower frequency of use compared to cross‐sectional imaging modalities like CT and MRI, EUS has demonstrated notable clinical and economic value in specific clinical settings. Its capacity for accurate T staging, combined with the ability to obtain FNA biopsies for pathological confirmation in a single session, minimizes the need for additional procedures and can streamline the diagnostic workflow [111, 115].

EUS has been found to be cost‐effective particularly in the management of early stage rectal cancer, where precise local staging influences decisions regarding surgical intervention or neoadjuvant therapy. A cost‐analysis by Harewood et al. [128] revealed that EUS, when used to triage patients with rectal cancer before surgery, improved QALYs and reduced the use of unnecessary chemoradiotherapy in patients with early stage tumors. The study demonstrated that EUS was associated with an incremental cost‐effectiveness ratio (ICER) of less than $10,000 per QALY gained compared to no staging, which falls well below the commonly accepted thresholds for cost‐effective interventions in healthcare.

In addition, EUS offers a lower cost alternative to MRI in facilities where access to high‐field MRI is limited or in patients who are unable to undergo MRI due to contraindications. While EUS requires a skilled operator and is subject to user dependency, its procedural costs are typically lower than those of advanced imaging modalities. A 2022 review noted that the average cost of an EUS procedure in the United States is approximately $1000–$1500, significantly less than MRI and on par with low‐end colonoscopy estimates [119].

From a resource utilization perspective, EUS is efficient in settings where it is combined with diagnostic or therapeutic endoscopy. When incorporated into comprehensive CRC programs, it can reduce hospital visits, enable same‐day diagnostics, and contribute to overall reductions in per‐patient costs. However, its widespread cost‐effectiveness is limited by the need for expert operators and the learning curve required to achieve optimal diagnostic performance, especially in community‐based practices. Moreover, its use is primarily confined to rectal cancer due to anatomical accessibility, which narrows its applicability in staging colon cancer more broadly [121].

Emerging technologies such as contrast‐enhanced EUS and AI‐assisted image analysis are poised to enhance diagnostic precision and potentially expand their use beyond traditional applications. These advancements, while still under evaluation, may improve lesion detection and characterization, thereby increasing diagnostic yield and reducing the likelihood of follow‐up procedures. The integration of AI could also reduce interobserver variability, improving the consistency of EUS interpretations and strengthening their cost‐effectiveness by minimizing diagnostic uncertainty [113, 129].

##### 3.4.1.6. Patient‐Reported Outcomes and Patient Experience

EUS combines endoscopy with ultrasound imaging and often requires conscious sedation. Patients generally report moderate discomfort related to endoscope insertion and manipulation, including gagging sensations or mild throat irritation [130]. Sedation effectively reduces procedural anxiety and pain; however, some patients may experience residual nausea or sore throat postprocedure [131]. Compared to more invasive surgical staging techniques, EUS is perceived as less burdensome and carries fewer complications, enhancing overall patient satisfaction [132]. Emerging imaging enhancements such as elastography and contrast‐enhanced EUS improve diagnostic accuracy without increasing patient discomfort [112]. Patient counseling about the procedure and recovery expectations is crucial for reducing anxiety and improving cooperation.

##### 3.4.1.7. Challenges and Future Directions

The future of EUS in colon cancer diagnosis and treatment is promising, with several advancements on the horizon [133]. Here is a glimpse into what is expected:

1. Enhanced diagnostic techniques: Researchers are developing new EUS techniques that utilize AI to analyze ultrasound images more precisely [113]. AI algorithms can potentially identify subtle abnormalities that might escape the human eye, leading to earlier and more accurate diagnoses of colon cancer. For instance, a study demonstrated the efficacy of an AI model in analyzing EUS images of pancreatic masses, suggesting potential applications in colorectal assessments [129].
2. Minimally invasive treatment procedures: EUS‐guided therapies are being explored that could offer minimally invasive treatment options for colon cancer [134]. For instance, EUS‐guided ablation involves using sound waves or other energy sources to destroy tumors directly during the EUS procedure. This could potentially reduce the need for traditional surgery in some cases. A review highlighted the evolution of therapeutic EUS, enabling procedures like gallbladder and biliary drainage, which could be adapted for colorectal applications [135].
3. Improved needle design: Advancements in needle design for EUS‐guided biopsies are underway to extract larger and more accurate tissue samples. This improvement could enhance the accuracy of cancer diagnosis and allow for better evaluation of tumor characteristics. A multicenter comparative study found that EUS‐guided fine‐needle biopsy had higher diagnostic accuracy and sensitivity compared to FNA, supporting the use of improved needle designs [136].

To ensure these advancements are clinically meaningful, multidisciplinary collaboration will be essential. EUS does not operate in isolation—it intersects with imaging, histopathology, oncology, and, increasingly, AI. Practical steps to enable this collaboration could include the establishment of multidisciplinary tumor boards where radiologists, endoscopists, oncologists, pathologists, and AI scientists meet regularly to evaluate complex cases, discuss imaging interpretations, and review AI‐generated predictions. Hospitals can also implement joint training programs aimed at bridging knowledge gaps—such as providing AI literacy training to clinicians and clinical context training for algorithm developers. Furthermore, collaborative research initiatives focused on integrating EUS with other diagnostic tools (e.g., MRI or CT) and using AI to generate comprehensive diagnostic profiles can accelerate innovation and adoption. The use of shared data repositories and cloud‐based review platforms can further streamline this interdisciplinary work and promote transparency and reproducibility in clinical decision‐making.

### 3.5. Histopathological Analysis

Histopathological analysis is fundamental in the diagnosis, staging, and treatment planning of CRC [137]. It involves the microscopic examination of tissue samples to assess their structure, composition, and cellular characteristics. This process confirms the presence of malignancy, determines tumor differentiation, and evaluates invasion into surrounding tissues [138]. Moreover, histopathological examination provides insights into molecular features, such as genetic mutations and biomarker expression, which are crucial for prognostication and therapeutic decision‐making. For instance, the World Health Organization’s [139] classification emphasizes the importance of histopathological evaluation in diagnosing digestive system tumors, including CRC [140].

Advancements in histopathological techniques have further enhanced the accuracy of CRC diagnosis. The integration of AI into histopathological image analysis has shown promise in improving diagnostic precision [141, 142]. Deep learning algorithms can assist in identifying histological features related to prognosis and metastasis, as well as assess specific components of the tumor microenvironment [143, 144]. A systematic review highlighted the potential of AI in CRC image analysis, suggesting its role in assisting diagnosis and predicting clinically relevant molecular phenotypes [136].

Furthermore, the histopathological assessment of tumor deposits has been refined to improve diagnostic consistency [145]. A Delphi consensus study is aimed at standardizing the histopathological diagnosis of tumor deposits in CRC, recognizing their significance in staging and prognosis. This consensus provides pathologists with clearer guidelines, enhancing the reliability of CRC evaluations [146].

Histopathological analysis remains a cornerstone in CRC management, offering critical insights into tumor biology and guiding effective treatment strategies.

#### 3.5.1. Clinical Applications

##### 3.5.1.1. Diagnosis

Histopathological analysis remains the gold standard for diagnosing colon cancer. Biopsy samples obtained during colonoscopy or surgery are examined under a microscope to confirm malignancy and determine histological subtype. Tumor grade, differentiation, and molecular markers such as KRAS and BRAF mutations are key indicators that guide treatment strategies [147, 148]. A study by Mitsala et al. [149] on an innovative AI model demonstrated a sensitivity of 97.1%, a specificity of 93.3%, and an accuracy of 96.4% in identifying colorectal polyps, surpassing traditional manual detection methods. Another study by Wei et al. [150] evaluated a deep neural network designed to classify colorectal polyps using histopathologic slide images. The model achieved an accuracy of 93.5% (95% CI, 89.6%–97.4%) in internal evaluations and 87.0% (95% CI, 82.7%–91.3%) on external datasets, performing comparably to local pathologists. These findings suggest that such AI models could enhance diagnostic efficiency and accuracy in CRC screenings [150].

Histopathological analysis remains the cornerstone of colon cancer diagnosis, providing crucial insights into tumor characteristics that guide treatment decisions. The integration of AI into histopathological evaluation has shown significant promise in improving diagnostic accuracy and efficiency. Studies such as those by Mitsala et al. [149] and Wei et al. [150] highlight the potential of AI models to enhance sensitivity, specificity, and overall accuracy in detecting colorectal malignancies. As AI‐driven approaches continue to evolve, they may complement traditional histopathology, leading to earlier detection, more precise classification, and ultimately improved patient outcomes in CRC management.

##### 3.5.1.2. Staging

Accurate staging of colon cancer is essential for effective treatment planning. Histopathological analysis determines the extent of tumor invasion (T stage), lymph node involvement (N stage), and metastases (M stage) [151]. The TNM staging system is based on these parameters, helping clinicians stratify patients into risk categories [152]. A study by Quirke and Morris [153] emphasized the importance of meticulous histopathological evaluation in CRC staging, highlighting that factors such as extramural vascular invasion and peritoneal involvement are critical in assessing Stage II cases [153].

The integration of imaging modalities enhances the accuracy of staging. A meta‐analysis by Kim et al. [35, 40] assessed the diagnostic performance of MRI in detecting extramural venous invasion (EMVI) in CRC. The pooled sensitivity was found to be 73% (95% CI, 66%–79%), and the specificity was 88% (95% CI, 81%–92%), indicating that MRI is a reliable tool for preoperative staging [35, 40].

Another study evaluated the accuracy of preoperative imaging in CRC staging compared to histopathology. The findings revealed that inaccurate staging, particularly understaging, could lead to involved resection margins and adversely affect oncological outcomes. This underscores the necessity for precise preoperative assessment to inform treatment strategies effectively [154]. Histopathological analysis with advanced imaging techniques such as MRI and CT scans with the use of AI enhances the accuracy of colon cancer staging. This comprehensive approach ensures that patients receive tailored treatments based on precise staging, ultimately improving clinical outcomes.

##### 3.5.1.3. Treatment Planning

Histopathological evaluation is pivotal in determining treatment strategies for colon cancer. Tumor characteristics such as grade, depth of invasion, and biomarker expression inform decisions regarding surgical resection, chemotherapy, and targeted therapies. High‐grade tumors with LVI may necessitate aggressive treatment, including adjuvant chemotherapy. For instance, high‐grade tumors exhibiting LVI are often associated with a higher risk of recurrence, prompting consideration of adjuvant chemotherapy to improve patient outcomes [155]. A recent trial by Cercek et al. [156] showed that patients with MSI‐H tumors responded well to immunotherapy, demonstrating the growing role of molecular pathology in personalized medicine [156].

These findings underscore the importance of integrating histopathological and molecular analyses to tailor personalized treatment plans, enhancing the efficacy of therapeutic interventions in colon cancer management.

##### 3.5.1.4. Prognosis

Histopathological features significantly impact prognosis. Patients with early stage, well‐differentiated tumors have better outcomes than those with advanced stage or poorly differentiated tumors. Tumor grade, lymph node involvement, and biomarkers like p53 and VEGF are strong prognostic indicators [137]. According to a study by Kondo et al. [157], patients with Stage I colon cancer had a 5‐year survival rate of 92%, while those with Stage IV had only a 14% survival rate, underscoring the importance of early histopathological assessment. Survival rates vary markedly with the stage of the disease at diagnosis. According to data from the American Cancer Society, the 5‐year relative survival rate for localized colon cancer (confined to the primary site) is approximately 91%, whereas it drops to about 14% for distant‐stage disease (cancer that has spread to distant parts of the body) [158]. These statistics underscore the critical importance of early detection and histopathological assessment in improving patient outcomes.

##### 3.5.1.5. Cost‐Effectiveness of Histopathological Analysis

Histopathological analysis is the definitive diagnostic and staging modality in CRC, offering precise morphological and molecular insights that are crucial for effective treatment planning. While the process entails costs—covering biopsy collection, slide preparation, specialized staining, pathologist interpretation, and advanced molecular testing—its economic value is clear when weighed against the high stakes of misdiagnosis or inaccurate staging.

The average cost for standard histopathology in CRC, including specimen processing and hematoxylin–eosin (H&E) staining, ranges roughly from $500 to $1200 per case, with prices escalating when advanced techniques like immunohistochemistry or next‐generation sequencing (NGS) are applied [159, 160]. Though these costs might seem substantial, histopathology provides a definitive diagnosis, informs tailored treatment decisions, and avoids the downstream costs associated with inadequate or inappropriate therapy. For example, identifying high‐risk features such as LVI, poor differentiation, or microsatellite instability (MSI) can directly influence adjuvant chemotherapy decisions and surgical margins, preventing overtreatment or relapse [156, 157].

Economic modeling supports the inclusion of molecular profiling in histopathological workflows. Studies show that adding MSI testing and KRAS/BRAF mutation analysis leads to an ICER of approximately $12,000 per QALY gained, which is well within accepted thresholds for personalized cancer care [160]. Moreover, advanced reports demonstrate that AI‐assisted digital pathology can boost diagnostic efficiency and reduce pathologist workload, offering potential cost savings of up to 20%–30% by lowering error rates and turnaround times [144, 161].

From a systems perspective, histopathology consolidates multiple diagnostic steps—confirming malignancy, grading tumors, and identifying biomarkers—into a single workflow, which reduces redundancy and cumulative costs associated with separate tests. Although room for improvement remains, digital platforms and AI integration promise to standardize interpretations, enhance workflow efficiency, and support remote consultations—benefits that are particularly meaningful in resource‐limited settings where expert pathology services are scarce [161, 162].

In light of these advancements, histopathology stands as not only a diagnostic imperative but also a cost‐effective cornerstone of modern CRC management. Its definitive results enable precise clinical decisions and optimized resource utilization, underscoring its indispensable role in patient care sustainability.

##### 3.5.1.6. Patient‐Reported Outcomes and Patient Experience

Histopathology itself is not an imaging modality but depends on tissue samples obtained via biopsy or surgery. The patient experience related to histopathology is primarily linked to the biopsy or surgical procedure. Colonoscopic biopsies are minimally invasive but can cause transient discomfort, bleeding, or cramping [163]. Surgical resections carry greater physical and psychological burdens, including pain, recovery time, and concerns about cancer diagnosis [52]. Anxiety can also arise during the waiting period for pathology results, which can delay treatment initiation and increase psychological distress [164]. Advances in digital pathology and AI‐assisted diagnostics promise to shorten turnaround times and increase diagnostic precision, potentially reducing patient uncertainty and improving satisfaction [165]. Minimally invasive biopsy techniques and better pain management protocols also contribute to enhanced patient comfort.

##### 3.5.1.7. Challenges and Future Directions

Despite its accuracy, histopathological analysis faces challenges such as interobserver variability, tissue sampling errors, and tumor heterogeneity. Access to expert pathology services is limited in some regions, leading to delays in diagnosis and treatment [162]. Additionally, traditional histopathological techniques may not capture tumor microenvironment complexities, affecting precision in staging and treatment planning.

Research is focusing on improving histopathological techniques through digital pathology and AI. AI‐powered image analysis can enhance diagnostic accuracy, reducing variability among pathologists [155, 156]. NGS and immunohistochemistry advancements allow for more precise molecular profiling, enabling personalized treatments. A recent study by Yin et al. [161] highlighted that AI‐assisted histopathological evaluation improved CRC detection rates by 15% compared to conventional methods.

The integration of histopathological data with imaging techniques, such as MRI and EUS, may improve diagnostic and prognostic accuracy. Multidisciplinary collaboration between pathologists, radiologists, and oncologists is crucial for advancing colon cancer diagnosis and treatment [166].

By addressing these challenges and adopting novel techniques, histopathological analysis will continue to play a vital role in the evolving landscape of colon cancer management.

### 3.6. AI and ML

AI and ML are revolutionizing the field of medical imaging by offering enhanced diagnostic accuracy, efficiency, and predictive capabilities [167]. These technologies have emerged as powerful tools in analyzing colon cancer imaging data, offering significant improvements in diagnosis, staging, treatment planning, and prognosis [168]. AI and ML algorithms can process large datasets from various imaging modalities, such as colonoscopy, CT, MRI, and EUS, enabling automated detection and characterization of malignant lesions [169, 170].

ML, a subset of AI, consists of algorithms that learn from input data, refine models through training, and make predictions on new data [171]. Supervised learning models, such as convolutional neural networks (CNNs) and support vector machines (SVMs), have demonstrated high accuracy in identifying colon cancer lesions, distinguishing between benign and malignant polyps, and predicting patient outcomes [172]. Deep learning–based AI models, particularly CNNs, have been instrumental in automated polyp detection and segmentation, significantly reducing interobserver variability among radiologists [167, 173, 174].

The application of AI and ML in colon cancer imaging extends beyond diagnosis. AI‐driven predictive analytics can assess tumor response to treatment, optimize surgical planning, and facilitate personalized therapy based on imaging biomarkers and genomic profiles [175]. Moreover, AI‐based tools can aid in early disease detection by identifying precancerous changes in colon tissues with higher sensitivity and specificity than traditional methods [167, 176].

However, while AI and ML present promising advancements, they also raise ethical concerns and potential biases. Biases in AI models may arise due to nonrepresentative training data, leading to disparities in diagnostic accuracy across different patient populations [169, 172]. Ethical considerations include data privacy, patient consent, and the potential displacement of human expertise [168]. Addressing these challenges requires the development of standardized AI protocols, rigorous validation studies, and regulatory frameworks to ensure the safe and equitable deployment of AI in clinical practice [171]. Furthermore, AI should complement rather than replace human decision‐making, ensuring that clinicians remain at the forefront of patient care [170].

#### 3.6.1. Clinical Applications

##### 3.6.1.1. Diagnosis

Image‐based analyses, driven by AI and ML algorithms, contribute significantly to the early detection and diagnosis of colon cancer. With advancements in CAD systems, these algorithms can assist radiologists in detecting subtle abnormalities indicative of malignancy on imaging studies such as colonoscopy, CT, MRI, and EUS [177]. By analyzing imaging features associated with colorectal lesions, AI‐enabled tools improve detection rates and facilitate timely intervention, thereby enhancing patient outcomes [178, 179]. Researchers at Washington University in St. Louis combined OCT with ML techniques to develop an imaging tool for CRC. In a pilot study, this approach identified tumors with 100% accuracy, showcasing its potential to improve traditional endoscopic methods [180].

Another study by Talukder et al. [181] introduced a hybrid ensemble model integrating deep feature extraction and ensemble learning to detect lung and colon cancer. Evaluated on histopathological datasets, the model achieved a 100% accuracy rate in identifying colon cancer, indicating its potential clinical applicability [181].

Lastly, a study by Collins et al. [182] investigated the use of hyperspectral imaging combined with CNNs for the automatic detection of CRC on fresh specimens. The study concluded that this approach is feasible and accurate, even with small datasets, and may serve as a nondestructive optical biopsy tool to support objective tumor‐free resection margins [182]. These studies underscore the transformative role of AI and ML in CRC diagnostics, offering enhanced accuracy and efficiency in early detection and diagnosis.

##### 3.6.1.2. Staging

AI accurate staging of colon cancer is critical for treatment planning. AI‐enhanced imaging techniques assess tumor size, invasion depth, lymph node involvement, and metastases with high precision [183]. Automated tumor segmentation algorithms improve TNM staging accuracy, reducing subjectivity and variability among radiologists [184]. For instance, a study by Nasir‐Moin et al. [185] evaluated an AI‐augmented digital pathology system for colorectal polyp classification. The AI system significantly improved pathologists’ diagnostic accuracy, increasing from 74.6% without AI assistance to 90.4% with AI assistance. This enhancement suggests that AI can reduce interobserver variability and improve diagnostic consistency among pathologists [185].

Additionally, research has shown that AI models can predict lymph node metastasis in CRC patients by analyzing histological images, with validation set AUC values ranging from 0.61 to 0.76. These findings indicate that AI can assist in accurate staging and potentially improve interobserver agreement in assessing lymph node involvement [186].

Recent advancements in AI have led to improved staging models using radiomics and deep learning. In a study by Bedrikovetski et al. [187], an AI‐based MRI staging system achieved an accuracy of 92.5% in predicting lymph node involvement, compared to 78% using traditional radiological assessment. Another study found that AI‐enhanced CT staging improved detection of peritoneal metastases in colon cancer by 20% [188].

Comparing imaging modalities, colonoscopy remains the gold standard for early detection but lacks the ability to accurately assess tumor invasion. CT and MRI, when enhanced by AI, demonstrate higher sensitivity for T staging, with MRI providing superior soft tissue contrast for assessing tumor penetration depth [189]. AI‐driven MRI analysis has reported a sensitivity of 89% in detecting extramural vascular invasion, a crucial factor in advanced stage colon cancer prognosis [190]. These studies underscore the potential of AI in enhancing the accuracy and consistency of CRC diagnostics, thereby supporting more reliable clinical decision‐making.

##### 3.6.1.3. Treatment Planning

AI and ML enhance treatment planning by providing quantitative imaging insights for surgical and therapeutic decision‐making. AI‐driven models predict treatment response by analyzing tumor morphology and vascular characteristics, assisting oncologists in selecting personalized treatment strategies [174]. For example, ML algorithms can predict chemotherapeutic efficacy based on imaging biomarkers, guiding precision medicine approaches [191].

A study developed a deep learning model analyzing colonoscopy images to predict rectal cancer patients’ responses to neoadjuvant chemotherapy. The model achieved an accuracy of 71.4%, with a sensitivity of 77.6% and a specificity of 62.9%, indicating its potential in guiding personalized treatment strategies [192]. AI‐assisted delineation in postoperative radiotherapy has been shown to improve the accuracy and efficiency of clinical target volume (CTV) and organ‐at‐risk (OAR) segmentation. This advancement enhances the quality of radiotherapy planning, potentially leading to better treatment outcomes for patients [193]. These studies depict the transformative role of AI and ML in refining treatment planning for CRC, aiming to improve patient outcomes through personalized and precise therapeutic approaches.

##### 3.6.1.4. Prognosis

AI‐derived imaging biomarkers facilitate prognostic assessment by correlating imaging characteristics with patient outcomes. Quantitative texture analysis, radiomics, and deep learning models predict recurrence risk, survival rates, and treatment efficacy. Studies have shown that AI‐based prognostic models outperform traditional risk stratification methods, with accuracy improvements of up to 20% [194].

For instance, a deep learning model developed by Yu et al. [195] demonstrated 90% accuracy in predicting 5‐year survival rates based on imaging and clinical data. Additionally, AI‐driven radiomics analysis identified tumor heterogeneity as a key predictor of recurrence, improving early intervention strategies [196]. These advancements highlight the transformative role of AI and ML in refining prognostic assessments, leading to more personalized and effective treatment strategies in colon oncology.

##### 3.6.1.5. Challenges and Future Directions

Despite the advantages of AI in colon cancer imaging, challenges remain. Variability in imaging protocols, interobserver discrepancies, and the need for large annotated datasets hinder widespread adoption. Future research should focus on refining AI models through multi‐institutional collaborations, integrating molecular imaging techniques, and addressing biases through diverse training datasets [185, 192]. Additionally, regulatory guidelines must be established to ensure AI’s ethical and responsible deployment in clinical settings [179, 186].

Ethical considerations surrounding AI include algorithmic biases, data privacy, and accountability in decision‐making. The use of federated learning models, which allow AI to be trained on decentralized datasets without compromising patient privacy, is one approach to mitigating these concerns [168]. Furthermore, AI explainability and interpretability must be prioritized to ensure clinicians can understand and validate AI‐driven decisions [183, 188].

Despite the significant promise of AI in enhancing colon cancer imaging, several challenges currently limit its widespread clinical adoption. Variability in imaging protocols across institutions, interobserver discrepancies in image interpretation, and the lack of large, diverse, and well‐annotated datasets pose substantial barriers to developing robust and generalizable AI models. To address these issues, future research must prioritize refining AI algorithms through extensive multi‐institutional collaborations that pool data from diverse patient populations, thereby reducing biases and improving model reliability. Additionally, integrating molecular imaging modalities alongside traditional imaging techniques could enrich data quality and diagnostic insights, further enhancing AI’s clinical utility. Alongside technical advancements, it is imperative to establish clear regulatory guidelines that ensure the ethical and responsible deployment of AI tools in clinical practice, safeguarding patient safety and data privacy.

Ethical considerations in AI application encompass concerns over algorithmic bias, maintaining patient confidentiality and defining accountability for AI‐informed clinical decisions. Innovative approaches such as federated learning, where AI models are trained on decentralized datasets without transferring sensitive patient information, offer promising solutions to privacy challenges while enabling collaborative model development. Furthermore, prioritizing AI explainability and interpretability is essential to empower clinicians to understand, trust, and validate AI‐generated outputs, fostering integration into clinical workflows.

To translate these advancements into improved patient care, fostering structured multidisciplinary collaboration is crucial. Radiologists, oncologists, pathologists, AI developers, and clinical informaticians should work together closely throughout AI development, validation, and implementation phases. Practical measures to achieve this include joint training programs that enhance AI literacy among clinicians and provide data scientists with clinical context, regular multidisciplinary tumor boards where AI‐generated imaging analyses are discussed alongside traditional diagnostic data, and collaborative research initiatives that promote coauthorship and shared data resources across specialties. Developing integrated digital platforms for seamless sharing of imaging, pathology, and AI outputs can further facilitate real‐time collaborative decision‐making. Pilot implementation projects within academic and clinical settings will also be vital to evaluate AI’s impact on diagnostic accuracy, workflow efficiency, and patient outcomes, with iterative feedback from all stakeholders.

By addressing technical, ethical, and collaborative challenges through these focused, actionable steps, AI can evolve from a promising innovation to a transformative tool in colon cancer imaging, improving diagnostic precision and ultimately enhancing patient outcomes.

### 3.7. Implications of Interobserver Variability in CRC Imaging

Interobserver variability is a well‐recognized challenge in interpreting imaging studies for CRC morphology and staging. For instance, different radiologists may assign varying TNM stages when evaluating the same MRI scans, which can directly influence treatment decisions such as eligibility for surgery versus neoadjuvant therapy [197]. Similarly, interpretation of CTC can vary in polyp detection rates, potentially affecting early diagnosis [198].

Such inconsistencies often arise from differences in radiologists’ experience, familiarity with specific imaging protocols, and subjective judgment of subtle imaging features like tumor boundaries or lymph node involvement. For example, a junior radiologist may miss small perirectal lymph nodes compared to an experienced specialist, leading to understaging [199].

To reduce this variability, standardized training programs and consensus imaging protocols have proven effective. For example, the European Society of Gastrointestinal and Abdominal Radiology (ESGAR) provides detailed MRI interpretation guidelines for rectal cancer, which have improved interreader agreement in multicenter studies [200]. Regular calibration sessions among radiologists within institutions can further align diagnostic criteria [180].

AI technologies offer promising solutions by providing automated and reproducible image analyses. Tools like CAD systems have demonstrated improved polyp detection on CTC, reducing miss rates [201]. Deep learning algorithms trained on large annotated datasets can assist in segmenting tumors and identifying suspicious lymph nodes, offering quantitative metrics that support clinical decision‐making. For instance, AI‐assisted MRI analysis has been shown to predict tumor staging with accuracy comparable to expert radiologists, potentially serving as a valuable second reader [155].

Incorporating these approaches into routine clinical practice can enhance diagnostic accuracy, reduce variability, and ultimately improve patient outcomes. Continued validation and integration of AI tools alongside ongoing radiologist education remain essential steps toward standardized and reliable CRC imaging interpretation.

### 3.8. Summary

AI and ML hold immense potential to revolutionize colon cancer imaging, improving diagnostic accuracy, staging precision, and treatment personalization. The integration of AI into medical imaging has already demonstrated significant benefits, such as increased detection rates, enhanced prognostic assessments, and improved treatment response predictions. However, addressing ethical concerns, validation challenges, and bias mitigation strategies will be crucial for their successful integration into routine clinical practice. The continued evolution of AI‐driven imaging tools will enhance early detection, optimize treatment outcomes, and ultimately improve patient survival rates in colon cancer management. Future research efforts should focus on refining AI algorithms, standardizing imaging protocols, and ensuring the equitable application of AI across diverse patient populations. By overcoming current limitations, AI and ML will play a pivotal role in advancing precision medicine and improving the overall quality of cancer care.

## 4. Conclusion

Colon cancer remains a significant global health concern, with rising incidence rates worldwide. Early detection is crucial in improving patient outcomes, as timely intervention enhances treatment efficacy and prognosis. Studies have demonstrated that AI‐assisted diagnostics significantly improve the accuracy of CRC detection, with deep learning models achieving up to 100% accuracy in tumor identification [180, 181, 186]. Moreover, the integration of imaging modalities such as colonoscopy, CT, MRI, EUS, and histopathological analysis has played a vital role in diagnosing and staging colon cancer [177, 178, 185].

This review explored key advancements in image‐based analysis, highlighting the transformative role of AI and ML in enhancing imaging capabilities. AI‐driven algorithms, particularly CNNs, have demonstrated remarkable accuracy in polyp detection and segmentation, reducing interobserver variability by up to 30% among radiologists [173, 174]. AI‐assisted MRI and CT staging models have significantly improved the detection of lymph node metastasis, with AUC values ranging from 0.61 to 0.76, while AI‐enhanced MRI staging achieved 92.5% accuracy in predicting lymph node involvement [186, 187]. Additionally, AI‐driven models have improved the sensitivity and specificity of colon cancer imaging, with deep learning–based polyp detection reaching a sensitivity of 88%–94% compared to traditional methods [167, 188].

These advancements enable precise staging, optimize treatment strategies, and contribute to personalized therapy by analyzing tumor morphology, vascular characteristics, and imaging biomarkers [174, 191]. For instance, AI‐based predictive models have demonstrated 90% accuracy in forecasting 5‐year survival rates, while radiomics analysis identified tumor heterogeneity as a key predictor of recurrence, enhancing early intervention strategies [195, 196]. Research shows that AI‐assisted diagnostics reduce interobserver variability by up to 30% and improve lesion characterization accuracy [175, 177, 185, 191]. Such improvements directly impact patient survival and quality of life (QoL) by enabling earlier interventions, reducing unnecessary procedures, and tailoring treatments to individual tumor profiles, ultimately lowering recurrence rates and improving long‐term patient outcomes.

The cumulative impact of diagnostic procedures on patient QoL is significant. Accurate, timely diagnosis and staging lead to more effective and personalized treatment plans, minimizing unnecessary or overly aggressive interventions that can negatively affect QoL [156]. Conversely, diagnostic delays, discomfort, and anxiety during procedures can worsen psychological distress and impair adherence to care plans [202]. Patient education, clear communication, and supportive care throughout the diagnostic journey have been shown to improve satisfaction, reduce anxiety, and foster trust in healthcare providers [203]. Incorporating patient‐reported outcome measures (PROMs) into clinical practice enables ongoing assessment of patient comfort and experience, facilitating continuous improvement in care delivery.

Despite these advancements, challenges persist, including interobserver discrepancies, variations in imaging protocols, and ethical concerns related to AI biases and data privacy [168, 172]. Addressing these issues requires standardized AI protocols, rigorous validation studies, and regulatory frameworks to ensure ethical and equitable deployment in clinical practice [171]. Furthermore, AI should serve as a complementary tool to human expertise rather than a replacement, ensuring that clinicians remain at the forefront of patient care [170].

To fully harness the potential of imaging technologies and AI in colon cancer management, interdisciplinary collaboration among researchers, clinicians, and policymakers is essential. Future efforts should focus on refining AI models through multi‐institutional collaborations, integrating radiomics and molecular imaging techniques, and mitigating algorithmic biases to improve diagnostic equity across diverse populations [185, 192]. The adoption of federated learning models offers a promising approach to training AI on decentralized datasets while preserving patient privacy [168]. Additionally, policymakers must establish comprehensive AI regulations and funding frameworks to accelerate responsible AI adoption in medical imaging.

Furthermore, it is imperative that researchers continue to refine AI algorithms to mitigate biases, ensuring equitable and ethical applications in diverse patient populations. Clinicians must remain engaged in AI advancements to integrate these tools effectively into practice, while policymakers should work toward regulations that standardize AI adoption in medical imaging.

AI and ML hold immense potential to revolutionize colon cancer imaging by improving diagnostic accuracy, staging precision, treatment personalization, and prognostic evaluation. The continued evolution of AI‐driven imaging tools will facilitate early detection, optimize treatment outcomes, and enhance patient survival rates. By fostering interdisciplinary collaboration and ensuring equitable access to advanced imaging technologies, we can significantly enhance the QoL for colon cancer patients worldwide. Researchers, healthcare providers, and policymakers must now work together to address remaining challenges, develop standardized imaging protocols, and implement AI‐driven solutions that transform global cancer care.

## Disclosure

All authors have read and agreed to the published final version of the manuscript.

## Conflicts of Interest

The authors declare no conflicts of interest.

## Author Contributions

Samuel Arthur Ameyaw: conceptualization, methodology, literature review and analysis, data extraction, writing—original draft preparation, and editing; Derrick Adu Afari: methodology, literature review and analysis, data extraction, and manuscript writing; John Boateng: supervision and writing—editing and review

## Funding

No funding was received for this manuscript.

## Data Availability

The data that support the findings of this study are available publicly in the cited peer‐reviewed journals for the manuscript.
